# Experimental Evidence of Buyang Huanwu Decoction and Related Modern Preparations (Naoxintong Capsule and Yangyin Tongnao Granule) in Treating Cerebral Ischemia: Intestinal Microorganisms and Transcriptomics in Rats

**DOI:** 10.1155/2022/4016935

**Published:** 2022-09-21

**Authors:** Junjun Yin, Jiehong Yang, Buchang Zhao, Chao Zhao, Wei Fu, Yu He, Miaolin Zeng, Yuting Yang, Xiaoyu Wei, Zhishan Ding, Jingxian Bai, Haitong Wan, Huifen Zhou

**Affiliations:** ^1^Zhejiang Chinese Medical University, Hangzhou, Zhejiang 310053, China; ^2^Buchang Pharmaceutical Co., Ltd., Xi'an, China; ^3^Department of Cardiac-Cerebral Diseases, Yinchuan Cardiac-Cerebral Treatment Internet Hospital, Yinchuan, China; ^4^Hangzhou Hongyu Pharmaceutical Technology Co. Ltd., Hangzhou 310053, China

## Abstract

**Background:**

The traditional Chinese medicines of Buyang Huanwu decoction (BYHW), Naoxintong capsule (NXT), and Yangyin Tongnao granules (YYTN) have excellent effects in preventing and treating cerebrovascular disease and are widely tolerated by patients. However, their effects on middle cerebral artery occlusion (MCAO) remain unknown.

**Methods:**

We evaluated gut microbiota alterations, the brain transcriptome, and nerve cell responses in rats with MCAO.

**Results:**

Our results showed that BYHW, NXT, and YYTN not only effectively improved the damaged state of blood vessels in rats and restored nerve function, but also improved survival. Additional experiments showed that treatment with BYHW, NXT, and YYTN regulated the intestinal microflora. Transcriptome analyses showed that BYHW, NXT, and YYTN modulated the transcriptome of rats with MCAO. The common mechanism of the three prescriptions for the treatment of cerebral ischemia may be related to the intestinal flora regulation of 60S ribosomal protein L18 (Rpl18), eukaryotic translation initiation factor 3 subunit, Ras homolog family member C, G protein subunit gamma 13 (Gng13), and Gng10 genes, among which Rpl18 is the most important. In addition, the three prescriptions had great specificity as anticerebral ischemia targets. Moreover, BYHW, NXT, and YYTN mitigated MCAO-induced hyperactivation of microglia and astrocytes.

**Conclusion:**

This study provides a foundation for further research on the mechanisms and treatment of IS. The results strongly suggest that key gut microbiota can be used to study functional genomics of brain, leading to novel discoveries about key genes involved in important biological processes.

## 1. Introduction

Stroke includes both hemorrhagic and ischemic cerebrovascular events. Approximately, 80% of incident strokes are characterized as ischemic stroke (IS, embolic or thrombotic strokes), and the remainder are characterized as hemorrhagic [1]. IS is the main cause of disability worldwide and remains difficult to treat. Intravenous tissue plasminogen activator and alternative treatment options such as intraarterial thrombectomy have complications and limitations [2]. Increasing evidence suggests that the microbiome-gut-brain axis is influenced by stress, while the gut microbiota can regulate stress-related responses. This phenomenon, reflected by altered intestinal microbiota and decreased diversity of gut-dwelling communities, is called dysbiosis. Dysbiosis promotes the systemic upregulation of inflammatory responses and has deleterious effects on acute IS [3, 4]. Manipulating gut microbiota is a promising therapeutic intervention for IS and thus has garnered increasing attention. Traditional Chinese medicine (TCM) regulates intestinal inflammation, either directly or indirectly, by modifying the gut microbiota [5]. Thus, gut microbiota plays an important role in IS [6].

TCM is widely used to treat IS due to its efficacy. Several empirical prescriptions for invigorating Qi and activating blood circulation have been developed, which have demonstrated efficacy in the clinic. Buyang Huanwu decoction (BYHW), Naoxintong capsules (NXT), and Yangyin Tongnao granules (YYTN) are important TCM formulas for cerebrovascular disorders [7–14]. According to TCM theory, brain activities depend on supplying the nutrients, Qi (energy), blood, Yin, and Yang [15–17]. BYHW, NXT, and YYTN (described in Tables 1–3) play important roles in treating IS such as tonifying Qi, invigorating blood, dredging collaterals, and nourishing Yin [10]. However, different drugs can be used to treat the same disease, showing that the mechanisms of action are complex. The underlying mechanisms are still a mystery. TCM has great potential to exert beneficial effects in IS by regulating gut microbes.

**Figure 1:**
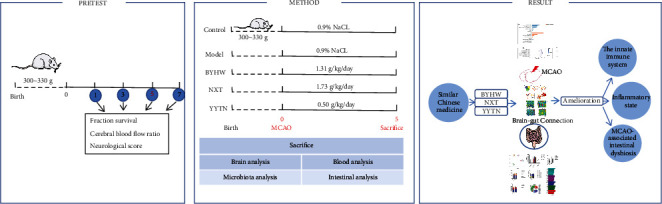
A flowchart of the study.

To explore the potential effects of TCM on IS and its underlying mechanisms, a rat model of middle cerebral artery occlusion (MCAO) was established. Rats were treated with the TCM formulas of BYHW, NXT, and YYTN on days 1, 3, 5, and 7 to treat MCAO. Survival rate and cortical blood flow levels were considerably ameliorated by day 5. Thus, test samples were acquired on day 5, and intestinal microflora in the intestinal tract and transcriptome gene in brain tissue were mainly detected. Our results suggest that BYHW, NXT, and YYTN can differentially affect the gut microbiota and transcriptome genome. Correlation analyses were conducted to identify key genes in the gut flora that can modify gene expression in brain regions. Meanwhile, abnormally activated immune cells attacked the central nervous system (CNS). A flow chart of the study was presented in Figure 1.

## 2. Materials and Methods

### 2.1. Animal and Drug Administration

SD male rats, weighing 280–320 g, were purchased from Shanghai Slacker Laboratory Animal Co., Ltd. (Shanghai, China), and housed at the Laboratory Animal Center of Zhejiang Chinese Medical Animal Care (AAALAC). Upon arrival, all the rats were acclimatized and fed for one week before starting the experiment. All rats were kept under standard environmental conditions (25 ± 1°C) with normal light/dark cycle conditions throughout the whole experiment.

### 2.2. Drugs

The specific ingredients of BYHW (2001621), NXT (Z20025001), and YYTN (2003L00206) are shown in Tables 1–3. Drug dosages were calculated based on the conversions from clinical adult dosages and the body weight of rat. According to our previous study, the BYHW group, YYTN group, and NXT group were administered by gavage with 1.31 g/kg/day BYHW, 0.5 g/kg/day YYTN, and 1.73 g/kg/day NXT.

### 2.3. Laser Speckle Imaging

The rats received anesthesia and then were placed lying prostrate on a unified fixed device. After shaving and disinfection of the operation areas, a longitudinal incision was made along the midline of the scalp, and the cranium was exposed. Next, the rats were placed in laser speckle imaging set-up system (MoorFLPI, Moor, UK). Data were acquired in panoramic radiography for a total of 5 min. Analysis was performed with moorFLPI V3.0 image. The blood flow after ischemia-reperfusion injury in rat brain was dynamically monitored at 1 d, 3 d, 5 d, and 7 d.

### 2.4. Sample Collection and 16S rRNA Sequencing

The feces of each group on day 5 were collected. The samples and their genomic DNA were immediately frozen in liquid nitrogen and then stored at −80°C. The total DNA was extracted from stool samples using the QIAamp DNA microbiome kit (Qiagen, Germany) according to the instructions manual. Gut microbiota 16S rRNA (V3-V4) of all fecal samples was amplified and measured using the Illumina MiSeq platform (Illumina HiSeq 2500, Illumina, USA).

The abundance and diversity of gut microbiota were analyzed. Clustering of samples was visualized using principal coordinate analysis (PC). The relative abundances were aggregated at each taxonomic rank: species, genus, family, order, class, and phylum. The characterization of microorganismal features differentiating the fecal microbiota was performed using the linear discriminant analysis (LDA) effect size (LEfSe) method (http://huttenhower.sph.harvard.edu/lefse/).

### 2.5. Transmission Electron Microscopy

Cerebral cortex of SD rats was separated and cut into 1 mm^3^. Samples were fixed with 2.5% glutaraldehyde solution overnight and then rinsed with 0.1 M phosphoric acid rinsing solution followed by 1% osmium acid at 4°C. The specimens were then dehydrated with a series of graded ethyl alcohols and pure acetone. For gradient infiltration with an embedding agent, the tissues were incubated with acetone solution (V/V = 1/1) for 1 h and in acetone solution (V/V = 3/1) for 3 h and kept away from light at room temperature overnight. The samples were treated with pure embedding agent overnight and heated to 70°C overnight to obtain a well-embedded sample. Then, 70–90  nm thick sections were cut. Ultrathin sections were generated for observation under a TEM (H-7650, Hitachi, Japan).

### 2.6. Scanning Electron Microscope

The tissues were fixed in glutaraldehyde for 1.5 h at room temperature (RT) and washed three times with buffer. They were then fixed in 1% ghrelin for 1 h and dehydrated with different concentrations of ethanol followed by dehydration with different concentrations of tert-butanol. Finally, the tissue was vacuum-dried for sectioning. Scanning electron microscopy (SEM) (S-3000N E-1010, Hitachi, Tokyo, Japan) was used to observe and take photographs.

### 2.7. Enzyme-Linked Immunoassay

Interleukin 18 (IL-18) (MB-1735A, Lot. No. 202105) in sera, IL-1*β* (MB-1588A, Lot. No. 202105) in sera, and short chain fatty acids (SCFAs) (MB-6918A) (all from Jiangsu Mei Biao Biological Technology Co., Ltd., Jiangsu, China) in feces were measured with an enzyme-linked immunoassay (ELISA) kit. All measurements were performed using an absorbance microplate reader (SpectraMax Plus384, Molecular Devices, San Jose, CA, USA).

### 2.8. Western Blot Analysis

Proteins (60 *µ*g) were separated on a 10% sodium dodecyl sulfate-polyacrylamide gel electrophoresis gel and electrotransferred to a nitrocellulose membrane (IPVH00010, Millipore, Burlington, MA, USA). After blocking in 5% milk at RT for 1 h, the membranes were incubated overnight at 4°C with the following primary antibodies: rabbit anti-60S ribosomal protein L18 (Rpl18) (1 : 2000, Cat. No. ab241988, Abcam, Cambridge, MA, USA), rabbit antieukaryotic translation initiation factor 3 subunit (Eif3c) (1 : 2000, Cat. No. 2068, Cell Signaling Technology (CST), Danvers, MA, USA), rabbit anti-Ras homolog family member C (Rhoc) (1 : 2000, Cat. No. 3430, CST), rabbit anti-G protein subunit gamma 13 (Gng13) (1 : 500, Cat. No. PA5-70258, Thermo Fisher Scientific, Waltham, MA, USA), rabbit anti-Gng10 (1 : 500, Cat. No. PA5-97046, Thermo Fisher Scientific), and mouse anti-*β*-actin (1 : 1000, Cat. No. ab8226, Abcam). Next day, membranes were incubated with secondary antibodies goat anti-rabbit IgG (H + L) (1 : 5000, Cat. No. 31210, Thermo Fisher Scientific) and goat anti-mouse IgG (H + L) (1 : 5000, Cat. No. 31431, Thermo Fisher Scientific) for 1 h. Proteins were detected by enhanced chemiluminescence.

### 2.9. Immunofluorescence

Brain tissue slides were blocked in 1% bovine serum albumin/phosphate-buffered saline for 1 h at RT and incubated with anticluster of differentiation 31 (CD31) (1 : 200, Cat. No. ab182981, Abcam), anti-ionized calcium binding adaptor molecule 1 (1 : 250, Cat. No. ab178847, Abcam), antiglial fibrillary acidic protein (1 : 400, Cat. No. ab49874, Abcam), and antineuronal nuclear protein (1 : 250, Cat. No. ab177487, Abcam) at 4°C for overnight, followed by incubation with corresponding secondary antibodies at RT for 1 h. The experiment was repeated three times with consecutive brain slices from each group, and the slides were observed by fluorescence microscopy in a blinded fashion.

### 2.10. Transcriptome

Tissue samples were lysed and total RNA was extracted by TRIzol reagent (15596018, Invitrogen, Carlsbad, CA, USA). RNA was purified by phenol: chloroform: isoamyl alcohol extraction followed by ethanol precipitation. mRNA was purified using oligo-dT beads (Qiagen, Hilden, Germany) and synthesized into cDNA, which was end-repaired, index adapter-ligated, and PCR-amplified. Library construction and RNA sequencing were done on the Illumina HiSeq 4000 platform, and the Agilent 2100 Bioanalyzer system was used to check their sizes. After sequencing, raw sequencing reads were processed into clean reads. Bowtie2 was used to compare clean reads to reference sequences, and RSEM was used to calculate the expression levels of genes and transcripts.

Based on the results of differential gene analysis, hierarchical clustering analysis was conducted with the pheatmap function in R software. Gene Ontology (GO) analysis was used to classify the functions of molecular function (MF), cellular component (CC), and biological process (BP) according to the results of differential gene analysis. The enrichment degree of GO terms was shown with a bubble plot, and the first 20 GO terms with the smallest *Q*-value were plotted. The functional classification of differential genes was conducted according to the results of GO annotation and official classification, and enrichment analysis was conducted with the phyper function in R software. The *P* value was calculated as follows: $P=1-\sum_{i=0}^{m-1}\left(\begin{array}{c} M\\ i \end{array}\right)\left(\begin{array}{c} N-M\\ n-i \end{array}\right)/\left(\begin{array}{c} N\\ n \end{array}\right)$.

Kyoto Encyclopedia of Genes and Genomes annotation results and official classification were used to classify the biological pathways of differential genes. The *P* value was further calculated and false discovery rate (FDR) was corrected. FDR ≤ 0.01 was considered as significant enrichment.

### 2.11. Statistical Analysis

All data were analyzed using SPSS 25.0 and plotted in GraphPad Prism 8.0 (GraphPad Prism software, San Diego, CA, USA). One-way analysis of variance was performed. All results are presented as the mean ± standard mean of error. *P* < 0.05 was considered statistically significant.

## 3. Results

### 3.1. Effects of BYHW, NXT, and YYTN on MCAO Survival Comparison and Cortical Blood Flow

**Figure 2:**
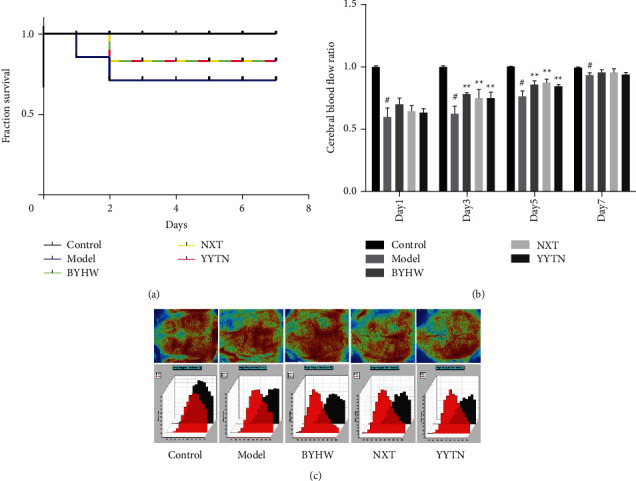
BYHW, NXT, and YYTN can ameliorate MCAO pathological characteristics effectively. (a) MCAO survival comparison in each group. (b) Statistical analysis about comparison of cerebral blood flow of day 1, day 3, day 5, and day 7. (c) Laser imaging of blood flow speckle in cerebral cortex on day 5. Compared with control group, ^#^*P* < 0.01; compared with MCAO model group, ^*∗*^*P* < 0.05, ^*∗∗*^*P* < 0.01, and *n* = 3/group.

To evaluate the effects of BYHW, NXT, and YYTN on MCAO rats, we monitored their survival rate and cerebral cortex laser speckle imaging. Mortality was higher in the model group; there were no significant differences among the BYHW, NXT, and YYTN groups (Figure 2(a)). At the same time, we monitored cortical blood flow laser speckle on days 1, 3, 5, and 7 after MCAO. From days 3 to 5, all three prescriptions significantly improved cerebral ischemic cortex blood flow recovery (*P* < 0.01). Rats also had strong self-healing ability after cerebral ischemia, and, on day 7, there was no significant difference between cerebral ischemic epidermis blood flow and the model group (*P* > 0.05, Figure 2(b)). Cephalic cortical blood flow on day 5 is shown in Figure 2(c). BYHW, NXT, and YYTN showed similar performance in ameliorating the survival rate and cortical blood flow.

### 3.2. Effects of BYHW, NXT, and YYTN on Microbial Community Structure

**Figure 3:**
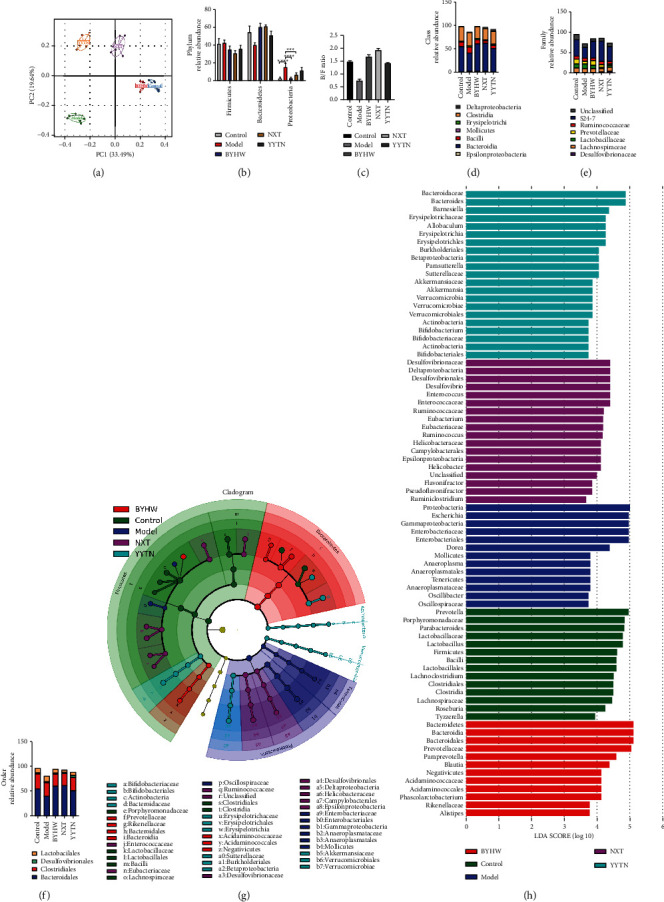
BYHW, NXT, and YYTN regulated the microbial community structure and key phenotypes. (a) Principal component (PC) analysis of the intestinal microbiota by taxonomic abundance patterns in rats. (b) Differences of gut microbiota at the phylum level. (c) Marker of dysbacteriosis of intestinal flora (Bacteroidetes*/*Firmicutes ratio). (d) Differences of gut microbiota at the class level. (e) Differences of gut microbiota at the family level. (f) Differences of gut microbiota at the order level. (g, h) LEfSe analysis between the control, the model, BYHW, NXT, and YYTN groups. Columns represent means ± SEM; ^*∗*^*P* < 0.05 and ^*∗∗*^*P* < 0.01.

There is growing evidence that intestinal dysbiosis is a key factor in stroke. Next, bacteria with the 16S v3-v4 DNA gene were sequenced to analyze the composition of the intestinal microbiota in normal and MCAO rats on day 5, and the effects of BYHW, NXT, and YYTN on the intestinal microbiota were evaluated. Principal component analysis (Figure 3(a)) showed that the microbiomes were structured differently among the control, MCAO, BYHW, NXT, and YYTN groups. The relative abundance of the predominant phyla in the five groups was compared to determine the structural changes in the gut microbiota (Figures 3(b), 3(d)–3(f)). At the phylum level, Firmicutes and Bacteroidetes are the most abundant bacteria in the gut microbiota and the ratio of Bacteroidetes to Firmicutes (B/F) is commonly used as marker of gut dysbiosis. Thus, the B/F ratio in the MCAO group was significantly lower than that in the other groups. Although there was no statistical significance, there was a clear trend. Treatment with BYHW, NXT, and YYTN significantly reversed the changes in the abundance of both phyla (Figure 3(c)). MCAO increased Firmicutes and Proteobacteria and reduced Bacteroidetes compared with the control. The trend of BYHW, NXT, and YYTN was closer to the control group (Figure 3(b)). At the class level, MCAO rats had high Bacilli and reduced Bacteroidia compared with the control (Figure 3(d)). However, this situation was ameliorated by BYHW, NXT, and YYTN treatment (Figure 3(d)). At the family level, MCAO showed a reduced proportion of *S24-7* compared with the control, which was significantly improved by BYHW, NXT, and YYTN treatment (Figure 3(e)). At the order level, MCAO reduced Bacteroidales compared with the control, and BYHW, NXT, and YYTN treatment led to improvements (Figure 3(f)). Linear discriminant analysis effect size analysis showed that 13 bacterial genera were depleted in MCAO (Figures 3(g) and 3(h)). It is worth noting that different representative bacterial genera were produced by BYHW, NXT, and YYTN treatment (Figures 3(g) and 3(h)). BYHW, NXT, and YYTN play different roles in gut microbial regulation. The ability of BYHW, NXT, and YYTN of altering intestinal microflora varied, depending on the different drug combinations.

**Figure 4:**
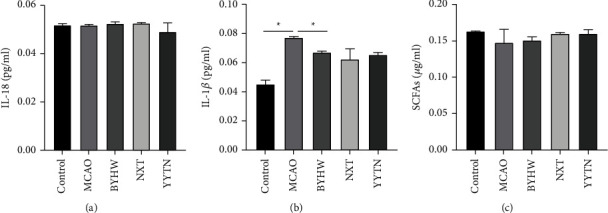
The expression of IL-18, IL-1*β* in sera (a, b), and SCFAs in feces (c). Data are expressed as the mean ± SEM; ^*∗*^*P* < 0.05.

Consistent with our expectations, SCFAs expression levels were reduced in MCAO. BYHW, NXT, and YYTN mildly upregulated SCFAs expression, but without statistical significance (Figure 4(c)). The expression of IL-18 and IL-1*β* was detected by ELISA. As shown in Figures 4(a) and 4(b), compared with MCAO, BYHW tended to reduce IL-1*β* expression (*P* < 0.05). Serum levels of IL-18 did not differ between the MCAO and treatment groups (BYHW, NXT, and YYTN).

### 3.3. Effects of BYHW, NXT, and YYTN on Transcriptome

**Figure 5:**
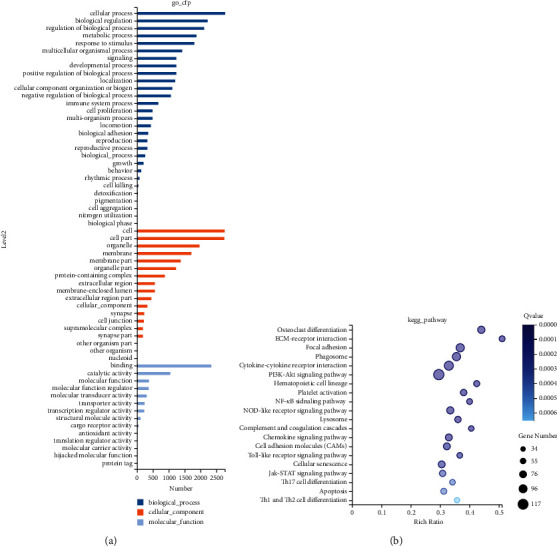
(a) The classification of component, process, and function (control and MCAO). The *y*-axis represents the Gene Ontology (GO) annotation terms, and the *x*-axis represents the number of total matched genes from a specific category. (b) KEGG pathway (control and MCAO). The *x*-axis shows enrichment score, and the *y*-axis indicates the KEGG pathway. The bubble size indicates the number of genes matched. The color of bubbles represents *q* value (the darker the color is, the smaller the *q* value is).

GO is divided into three major functional categories: BP, CC, and MF. For the biological process, the sham and MCAO groups were mainly enriched in the categories of cellular process, biological regulation, regulation of biological process, metabolic process, and response to stimulus. The cellular components primarily involved the cytoplasm, membrane, organelles, and membrane parts. The enriched molecular function gene sets mainly involved binding and catalytic activity (Figure 5(a)). KEGG pathway enrichment analysis identified these differentially expressed genes (DEGs) between sham and MCAO groups which are shown in Figure 5(b) detailed as follows: osteoclast differentiation, extracellular matrix- (ECM-) receptor interactions, focal adhesions, phagosomes, cytokine-cytokine receptor interactions, PI3K-Akt signaling, platelet activation, the hematopoietic cell lineage, NF-*κ*B and Nod-like receptor signaling pathways, Lysosomes, complement and coagulation cascades, Toll-like receptor pathway, chemokine signaling, cell adhesion molecules (CAMs), Jak-STAT pathways, Th17 cell differentiation, cell apoptosis, and Th1/Th2-cell differentiation. BYHW, NXT, and YYTN behaved similarly (Supplementary Figures 1–3). GO annotation and KEGG analysis between MCAO and BYHW group were shown in Supplementary Figure 1. GO annotation and KEGG analysis between MCAO and NXT group were shown in Supplementary Figure 2. GO annotation and KEGG analysis between MCAO and YYTN group were shown in Supplementary Figure 3.

### 3.4. The Correlation Analysis of BYHW, NXT, and YYTN on Intestinal Flora and Critical Transcriptional Genes

**Figure 6:**
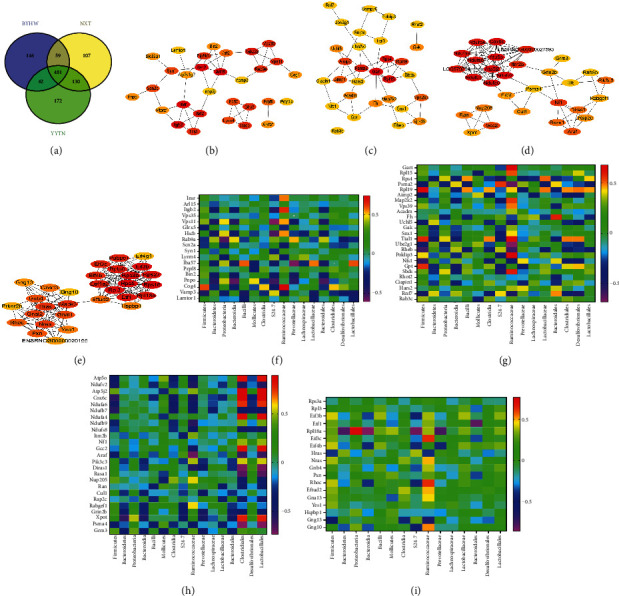
(a) Venn diagram. 481 genes are coregulated by BYHW, NXT, and YYTN. There are 146 genes specifically regulated by BYHW; 107 genes are specifically regulated by NXT and 172 genes are specifically regulated by YYTN. (b–e) Analysis of protein interaction network by STRING. The first 30 genes of BYHW specifically regulated are shown in (b). The first 30 genes of NXT specifically regulated are shown in (c). The first 30 genes of YYTN specifically regulated are shown in (d). The first 30 coexpressed genes of BYHW, NXT, and YYTN are shown in (e). (f–i) Heatmaps of correlation between characteristic intestinal flora and key genes. (f) BYHW-based heatmap; (g) NXT-based heatmap; (h) YYTN-based heatmap; (i) BYHW, NXT, and YYTN-based heatmap.

The group between control, MCAO, BYHW, NXT, and YYTN gene interactome was analyzed by STRING (https://string-db.org/) to reveal gene-gene connections. The PPI network was constructed using DEGs based on STRING PPI confidence scores >0.4 in the STRING platform. Our results show that there was a trend of reversal of 1157 subclass-specific trait genes, including 146 BYHW-specific genes, 107 NXT-specific genes, 172 YYTN-specific genes, and 481 genes coreversed by BYHW, NXT, and YYTN (Figure 6(a)). We further selected 30 genes from the PPI network identified by transcriptomics (Figures 6(b)–6(e)) and then explored correlations between gene expression (differential expression) and some identified gut microbiota genera (Figures 6(f)–6(i)). As we can see in Figure 6(f) (the genes specific reversal in BYHW), the Cog4 was associated with Firmicutes, with a correlation close to 0.6; the Rab9a was associated with Proteobacteria, with a correlation close to 0.557; the Pnpo was associated with Bacteroidia and *S24-7*, with correlation close to −0.568 and −0.518, respectively; the Iba57 was associated with Bacilli, with a correlation close to 0.579; the Insr had a negative correlation with Ruminococcaceae, and the corresponding correlation coefficients was −0.557; the Itgb2, Vps11, and Hscb had a positive correlation with Ruminococcaceae, and the *R* values were 0.636, 0.525, and 0.536. Strong positive correlation was found between Cog4 and Ruminococcaceae, with a correlation close to 0.743. The Rab9a was associated with Prevotellaceae, with a correlation close to 0.514; the Iba57 was associated with Lactobacillaceae and Lactobacillales, with correlation close to 0.557 and 0.589; the Pnpo was associated with Bacteroidales, with a correlation close to −0.536. In NXT, the Rpl19, Tial1, Rheb, Nfs1, and Gpt were associated with Firmicutes. The corresponding correlation coefficients were 0.661, 0.575, −0.589, −0.561, and 0.564, respectively. Psma2 was associated with *S24-7*, with a correlation close to 0.55; Fh was associated with Bacteroidales, with a correlation close to 0.591. In YYTN, the Cox6c and Ndufb7 were associated with Firmicutes, with correlation close to −0.568 and −0.554. Xpot was associated with Bacteroidetes and Proteobacteria, with a correlation close to −0.589 and 0.521. The Ndufv2, Ndufb9, Araf, and Diras1 had a negative correlation with Ruminococcaceae, with correlation coefficients of −0.521, −0.571, −0.7, and −0.539, respectively. The Nup205 and Rabgef1 had a positive correlation with Ruminococcaceae. The corresponding correlation coefficients were 0.561 and 0.575, respectively. Eventually, our results suggest that the reversion trend between BYHW, NXT, and YYTN had a significant number of common genes. Gng13 was associated with Firmicutes, with a correlation close to −0.532. Rpl18a was associated with Bacteroidetes, Proteobacteria, Bacteroidia, and *S24-7.* The corresponding correlation coefficients were −0.771, 0.761, −0.857, and −0.839, respectively. The Eif3c, Rhoc, and Gng10 had a positive correlation with Ruminococcaceae, with correlation coefficients of 0.625, 0.625, and 0.521. The Gng13 was associated with Ruminococcaceae, with a correlation close to −0.632. The Rpl18a was associated with Bacteroidales, with a correlation close to −0.807.

### 3.5. Effects of BYHW, NXT, and YYTN on Key Genes

**Figure 7:**
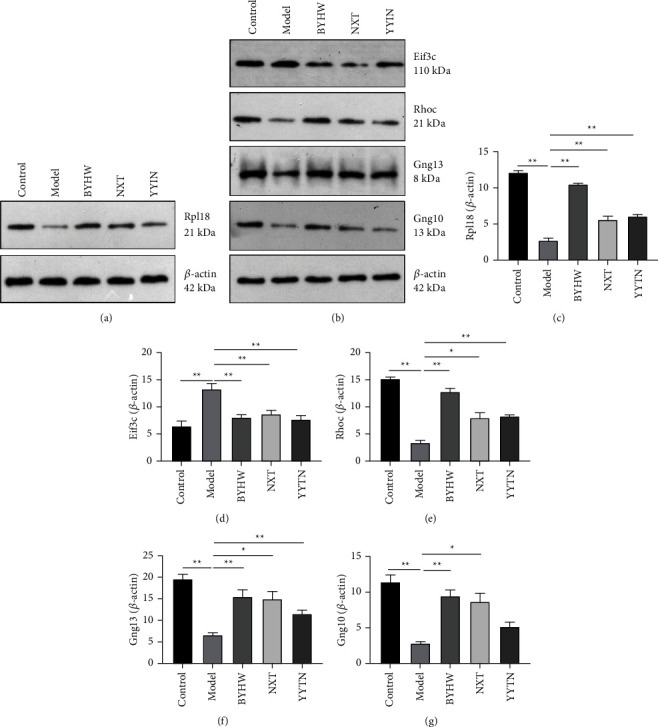
BYHW, NXT, and YYTN can reverse the expression of key genes. (a–g) The proteins levels of Rpl18, Eif3c, Rhoc, Gng13, and Gng10. Representative images of WB experiments are shown. Data are expressed as the mean ± SEM; ^*∗*^*P* < 0.05, ^*∗∗*^*P* < 0.01, and *n* = 3/group.

To determine whether BYHW, NXT, and YYTN share a common protein mechanism involved in the treatment of MCAO, Rpl18, Eif3c, Rhoc, Gng13, and Gng10 was detected by western blot analysis (Figures 7(a)–7(g)). The expression of Rpl18, Rhoc, Gng13, and Gng10 proteins all increased in response to BYHW, NXT, and YYTN treatment. In addition, Eif3c expression was decreased by BYHW, NXT, and YYTN treatment in MCAO. These data suggest that the common therapeutic effects of BYHW, NXT, and YYTN may be related to their ability of differentially regulating some common key genes.

### 3.6. Effects of BYHW, NXT, and YYTN on Endodermal Ultrastructure and Cerebral Microvessels

**Figure 8:**
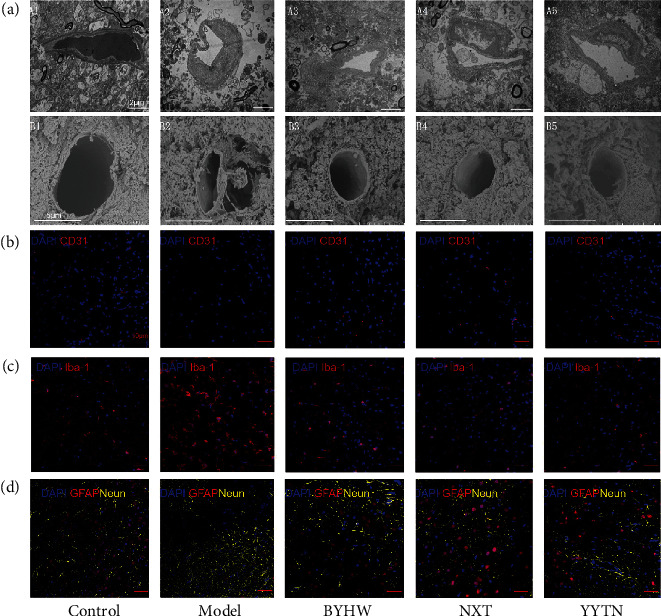
Effects of BYHW, NXT, and YYTN on endodermal ultrastructure, cerebral microvessels, and neuroimmune cell on the 5th day after reperfusion. (a) Representative images of ultrastructure under TEM (up, bar = 2 *μ*m) and SEM (down, bar = 5 *μ*m). (b) Representative images showing pathological features among the endothelial cells. Bar = 50 *μ*m, *n* = 3. (c) Representative images of pathological sections of the microglia in brain tissue in different groups are shown. Bar = 50 *μ*m, *n* = 3. (d) Representative images of the astrocytes and neurons in brain tissue on 5th day after reperfusion. Bar = 50 *μ*m, *n* = 3.

Each microstructure of the microvessels was clearly visualized by transmission electron microscopy. Compared with the control, MCAO not only has a narrow lumen and rough surface, but also has few organelles (Figure 8(a)). Vascular insults were significantly positively regulated by BYHW, NXT, and YYTN (Figure 8(a)). Morphological changes were evaluated by SEM. The MCAO group had a significantly larger area for capillaries than the control group. The MCAO showed a significantly larger area of capillary pericyte constriction than the control group (Figure 8(a)). In parallel, the perivascular lesions were loosened, and foam-like vacuoles were found along the lesions. As can be seen from Figure 8(a), BYHW, NXT, and YYTN treatment effectively reversed the damaged state of microvessels. CD31 is a marker of the vascular endothelium. Immunofluorescence results showed that brain microvascular endothelial cells (ECs) were discontinuous in MCAO, whereas BYHW, NXT, and YYTN treatment showed continuous vessels. These data suggest that BYHW, NXT, and YYTN can protect the ECs (Figure 8(b)).

### 3.7. Effects of BYHW, NXT, and YYTN on Neuroimmune Cells

Neurons are the main bearers of normal nervous system functions. The majority of astrocytes contact blood vessels, and astrocytes are reliant on trophic support from the vasculature. After establishing a model of cerebral ischemia injury in rats, the number of neurons decreased, resulting in an incomplete structure of the nervous system and further loss of function (Figure 8(c)). Hyperproliferation of astrocytes and formation of glial scarring can affect normal local neural circuits, which is detrimental to neurogenesis. Activated microglia release different cytokines, which causes deterioration of the inflammatory. In this study, BYHW, NXT, and YYTN mitigated MCAO-induced hyperactivation of microglia and astrocytes to different extents (Figures 8(c) and 8(d)).

## 4. Discussion

BYHW is a well-known formula by WANG Qing-ren of the Qing Dynasty. NXT (national medicine permission number: Z20025001) is derived from the classic formula BYHW. It has gained the National Chinese Medicine Protection Certificate in 2014 and has been included in the National Basic Drug List (2012 edition) and the Chinese Pharmacopoeia (2015 edition) [11]. YYTN, similar to BYHW and NXT prescriptions, is a modern TCM developed by professor Wan Hai-Tong who successfully obtained a patent and approval from the China Food and Drug Administration (approval no. 2003L00206). They all reportedly displayed antistroke properties at the cellular, animal, and human level [7, 9, 10, 14, 18–20]. A clinical protocol on BYHW, NXT, and YYTN is being evaluated [10]. In this study, the potential role of gut microbiota in the development of IS was assessed from animal experiments, and the results provide new laboratory evidence for the clinic. We first demonstrated that treatment of the MCAO rat model with BYHW, NXT, and YYTN for 5 days significantly improved the survival rate and neurological functional score and reduced the cerebral infarct area to different degrees. Our results showed that BYHW, NXT, and YYTN may exert therapeutic effects by regulating the composition of gut microbiota, inhibiting the production of proinflammatory factors, and promoting the generation of SCFAs. These changes might have a different immune status in the neuroimmune cells of IS. Several mechanisms may underlie the association between gut microbiota and neuroimmune cells.

In the MCAO model, a microbiome imbalance will influence the outcome of IS by inhibiting the transport of effector T cells from the gut [21]. IS is associated with different microbiota structures [22]. IS patients show significant dysbiosis of the gut microbiota with enriched SCFAs producers, including *Odoribacter* and *Akkermansia* [4]. Singh et al. [23] showed reduced species diversity and bacterial overgrowth of Bacteroidetes and in turn effects on stroke outcome via immune-mediated mechanisms. Reports have also claimed that the *F/B* ratio might be one of the key and essential criteria for diagnosing diarrhea in animals [24]. Dominant bacteria at the class level were Bacilli, Clostridia, and Gammaproteobacteria in the ileum and Bacteroidia and Clostridia in the colon and cecum. Bacteroidales, Enterobacteriaceae, Clostridia, Lactobacillales, and *Turicibacter* in the ileum and Bacteroidales and Clostridia in the colon and cecum were the dominant bacteria at the order level. *S24-7*, a dominant family belonging to the class Bacteroidales, degrades complex polysaccharides into acetate, propionate, and succinate. As shown in this study, BYHW, NXT, and YYTN significantly changed the gut microbiome structure and modulated the composition of gut microbiomes at different levels. We found differences in the microbiome, between MCAO and TCM (BYHW, NXT, and YYTN), including in Firmicutes, Bacteroidetes, and Proteobacteria at the phylum level, Bacilli and Bacteroidia at the class level*, S24-7* at the family level, and Bacteroidales at the order level. Meanwhile, SCFA is a major class of intestinal microbial groups that affects body immunity. Beneficial effects of SCFAs in colitis and metabolic syndrome have been reported, which manifest as maintaining intestinal integrity and exerting anti-inflammatory effects [25]. In addition, it has been suggested that *Ackermann* might play a key role in healing wounds by promoting butyrate levels, thus consolidating the integrity of epithelial cells in poststroke mice [26]. In our study, BYHW, NXT, and YYTN slightly enhanced the levels of SCFAs in feces. These results suggest that the intestinal flora with SCFAs may be involved in the stroke and be correlated with the severity of IS.

A growing body of research has been instrumental in showing that microbiota play a crucial role in fundamental neural processes such as neurodevelopment, neuroinflammation, and behavior [27–31]. Some studies in germ-free mice have shown that, to some extent, the maturation and function of microglia depend on the composition and SCFA regulates microglia homeostasis [32, 33]. In addition, the indirect effects of gut microbiota on the innate immune system may lead to changes in circulating levels of proinflammatory and anti-inflammatory cytokines, which directly affect the function of microglia. Moreover, similar to microglia, overactivation of astrocyte can lead to the production of neurotoxic substances or immune inflammatory substances. The metabolites of gut microbiota stimulate astrocyte activation and inhibit the recruitment and ability of neurotoxic immune cells by participating in the type I interferon (IFN) and aryl hydrocarbon receptor axis [34]. In our study, different groups of BYHW, NXT, and YYTN inhibited the abnormal activation of microglia and astrocyte to varying degrees, probably reflecting the effects of TCM-gut microbes-gliocyte-stroke. Glial cell and neurons are cell populations that are directly targeted by IFN-*β* to arrest CNS inflammation [34–37]. Therefore, we presume that BYHW, NXT, and YYTN regulate inflammatory factors through gut microbes, which in turn affect neuroglia.

The body regulates the functions of brain and intestine through the bidirectional loop of the brain-intestine axis, which is the hub of the interaction between the brain and the gastrointestinal tract. Gut flora plays an important role in the brain-gut interaction. It not only participates in the decomposition, digestion, and absorption of fats, amino acids, and sugars, but also plays an important role in human health [38]. Moreover, gut microbes can resist the invasion of pathogens through their immune function, thus promoting the stability of host regulatory systems. Changes in gut microbiota homeostasis can affect hormones, immunity, and vagus nerve pathways in the gut and brain, as well as affecting brain genes in humans and animals, such as bacterial fermentation products and gut endocrine factors, thereby regulating brain function. We further identified key genes in the brain transcriptome (Rpl18, Eif3c, Rhoc, Gng13, and Gng10), which may be associated with key gut microbiota. These genes may be the common mechanism of the TCM formulas in treating cerebral ischemia through the brain-gut axis. Rpl18 is an important component of the large subunit of ribosome and plays an important role in ribosome assembly. C Chen [39] reported that Rpl18 regulates erythroid maturation via JAK2/STAT3 signaling. Loh JT [40] demonstrated that suppressing JAK2/STAT3 signaling in neutrophils was important for intestinal homeostasis. In this study, BYHW, NXT, and YYTN increased the expression of Rpl18. The regulation of “Rpl18-JAK2/STAT3-intestinal homeostasis” may be a critical way for BYHW, NXT, and YYTN to treat IS. Eif3c, the core subunit of eukaryotic translation initiation factor 3, was associated with abnormal protein translation, which can inhibit apoptosis. In mice, Fujii et al. found that Eif3c was required for Shh-mediated tissue patterning and Eif3c might have a unique sensitivity to the Shh receptor patched 1 (Ptch1) dosage [41]. In another study, inflammatory diseases of the gut gave rise to dramatic increases in epithelial Shh signaling [42], and Shh signaling could control gut epithelium homeostasis [43]. In addition, Eif3c may play a significant role in the development of colon in mice [44]. In this study, we found that the expression of Eif3c decreased in MCAO. The regulation of “Eif3c-Shh-intestinal homeostasis” might be one of mechanisms for BYHW, NXT, and YYTN to treat IS. Rhoc GTPase is a critical regulator of Fc*γ*R-mediated phagocytosis in macrophages [45]. Additionally, Fc*γ*R-mediated phagocytosis could induce proinflammatory cytokine production in murine macrophages [46]. Furthermore, Rhoc has been reported to be involved in regulating cytoskeletal reorganization and affecting cell adhesion and migration [47]. In this study, the regulation of “Rhoc-inflammatory-cell adhesion” may be a way for BYHW, NXT, and YYTN to treat IS. Beside the above genes, several G proteins have also been noticed. The abnormal secretion of intercellular transmitters and hormones is one of the important features of neuroendocrine cells, regulated by G protein-coupled membrane receptors [48]. Gng10 encodes a subunit of G protein that is involved in the regulation of the cell cycle. Studies have shown that overexpression of Gng10 promotes the progression of colorectal cancer [49]. Some additional points deserve to be discussed. For instance, Gng13 may regulate the lymphangiogenesis [50], and it may be a potential marker of Alzheimer's disease [51]. However, the roles of Rpl18, Eif3c, Rhoc, Gng13, and Gng10 in IS have not been reported. Studies have shown that BYHW, NXT, and YYTN inhibit the inflammatory factors [52, 53]. Any of the factors discussed above may be relevant. Accordingly, we hypothesize that BYHW, NXT, and YYTN exert protective effects by improving the structure of the flora and its intestinal homeostasis and ameliorating systemic hypoinflammation, further affecting the immune response of host cells and regulating the expression of key genes in the brain. Rpl18, Eif3c, Rhoc, Gng13, and Gng10 may also be regulated by BYHW, NXT, and YYTN. BYHW, NXT, and YYTN are targets for anti-inflammatory effects through regulation of the intestinal flora. Although speculative, this could also shed some light on why different herbs can alleviate MCAO symptoms. We interpret these findings as indicating possible homoeopathic and heterogeneous treatments.

The innate immune system is one of the first lines of defense against invading microorganisms, and evidence about the function of microbes on the brains innate immune system is constantly increasing. Increasing evidence [54–56] about the brain-gut axis has been reported, indicating the possibility of therapeutic advances in disease treatment through modulation of the structure of the gut microbiota, the metabolites of intestinal bacteria, and intestinal mucosal barrier. However, based on the current state of our knowledge, the role of the microbiota in moderating stroke is just beginning to be understood. Few standardized IS treatment protocols exist. Experimental models have been essential for the TCM-microbiota-gut-brain research. This is the first study to show a similar tendency of intestinal flora in MCAO rats with invigorating *Qi* and activating blood circulation prescriptions (BYHW, NXT, and YYTN). BYHW, NXT, and YYTN effectively restore the structure of the intestinal flora, increase the expression of SFCA, and inhibit the overexpression of glial cells, revealing a possible immune mechanism underling these clinical prescriptions.

Our study is unique and innovative in the fact that it provides a multidimensional view of the modern theoretical support for TCM treatment. In the future, it would be interesting to explore how gut microbiota affects the immune cells of brain. Intestinal microbiota on the expression of genes signifying regulatory immunity may be possible. However, this study also had some limitations. The analysis between intestinal flora imbalance and transcription has not been rigorous enough. Clinical cases should be included, and more experiments should be performed to verify these connections.

## 5. Conclusion

In the present study, our work highlighted the therapeutic effect of BYHW, NXT, and YYTN (traditional Chinese medicine about tonifying qi and activating blood) on MCAO. Additionally, the transcript levels for various genes of brain were evaluated by RT-qPCR and gut bacterial communities were investigated with 16S rRNA gene amplicon sequencing. And BYHW, NXT, and YYTN significantly changed the gut microbiomes structure and brain genes at different levels. The protective effect of BYHW, NXT, and YYTN on brain nerves was studied from the perspective of the microflora-brain-gut axis. Together, these data provide further evidence that BYHW/NXT/YYTN can treat MCAO, but their specific targets are different.

## Figures and Tables

**Table 1:** The specific ingredients of BYHW.

Chinese name	Latin name	Weight^1^ (g)
Huang Qi	*Hedysarum multijugum Maxim*	60
Tao Ren	*Persicae semen*	9
Hong Hua	*Carthami flos*	9
Chi Shao	*Radix Paeoniae Rubra*	9
Dang Gui	*Angelicae sinensis radix*	9
Chuan Xiong	*Chuanxiong Rhizoma*	6
Di Long	*Lumbricus*	9

^1^It was the preparation of affiliated hospital (2001621), 10 g/bags.

**Table 2:** The specific ingredients of NXT.

Chinese name	Latin name	Weight^2^ (g)
Huang Qi	*Hedysarum multijugum Maxim*	66
Tao Ren	*Persicae semen*	27
Hong Hua	*Carthami flos*	13
Chi Shao	*Radix Paeoniae Rubra*	27
Dang Gui	*Angelicae sinensis radix*	27
Chuan Xiong	*Chuanxiong Rhizoma*	27
Ru Xiang	*Olibanum*	13
Mo Yao	*Myrrha*	13
Dan Shen	*Radix Salviae*	27
Niu Xi	*Achyranthis bidentatae radix*	27
Di Long	*Lumbricus*	27
Gui Zhi	*Cinnamomi ramulus*	20
Sang Zhi	*Ramulus Mori*	27
Ji Xue Teng	*Spatholobus suberectus Dunn*	20
Shui Zhi	*Hirudo*	27
Quan Xie	*Scorpio*	13

^2^The abovementioned drugs were carefully ground to pass through the 80 mesh sieve and then fill the capsules (amount 0.4 g per grain).

**Table 3:** The specific ingredients of YYTN.

Chinese name	Latin name	Weight^3^ (g)
Huang Qi	*Hedysarum multijugum Maxim*	1360
Chuan Xiong	*Chuanxiong Rhizoma*	910
Shui Zhi	*Hirudo*	310
Ge Gen	*Radix Puerariae*	1640
Shu Di Huang	*Rehmanniae Radix Praeparata*	1360
Shi Hu	*Herba Dendrobii*	910

^3^All drugs were made of 1000 g particles, 9 g/bags.

## Data Availability

The datasets used during this study are available from the corresponding author upon reasonable request.

## Abbreviations

BYHW: Buyang Huanwu decoction
NXT: Naoxintong capsule
YYTN: Yangyin Tongnao granule
TCM: Traditional Chinese medicine
MCAO: Middle cerebral artery occlusion
TPA: Tissue plasminogen activator
CNS: Central nervous system.

## Supplementary Materials

Supplementary Materials Supplementary_Figure_1: (a) The classification of component, process, and function (MCAO and BYHW). The *y*-axis represents the Gene Ontology (GO) annotation categories and the *x*-axis represents the number of total matched genes from a specific category. (b) KEGG pathway (MCAO and BYHW). *X* axis is enrichment score, and the *y*-axis indicates the KEGG pathway. The bubble size indicates the number of genes matched in the KEGG pathway. Supplementary_Figure_2: (a) The classification of component, process, and function (MCAO and NXT). (b) KEGG pathway (MCAO and NXT). Supplementary_Figure_3: (a) The classification of component, process, and function (MCAO and YYTN). (b) KEGG pathway (MCAO and YYTN).
