# Supplementary figures and images for: Experimental Evidence of Buyang Huanwu Decoction and Related Modern Preparations (Naoxintong Capsule and Yangyin Tongnao Granule) in Treating Cerebral Ischemia: Intestinal Microorganisms and Transcriptomics in Rats
Source: Evid Based Complement Alternat Med. 2022 Sep 21;2022:4016935. doi: 10.1155/2022/4016935 (PMC9519341; doi:10.1155/2022/4016935)

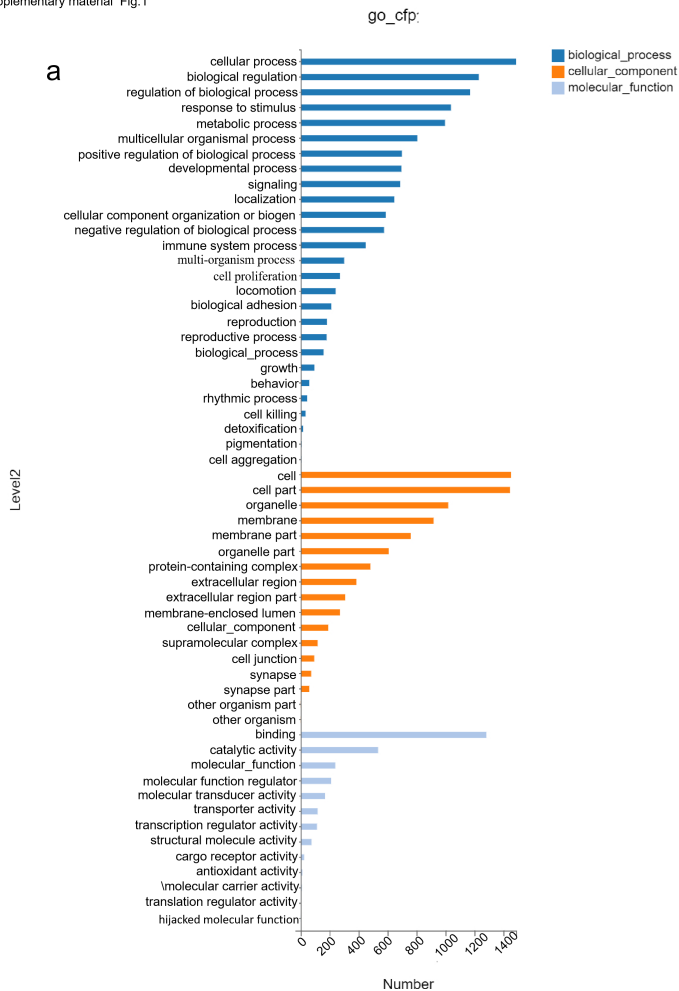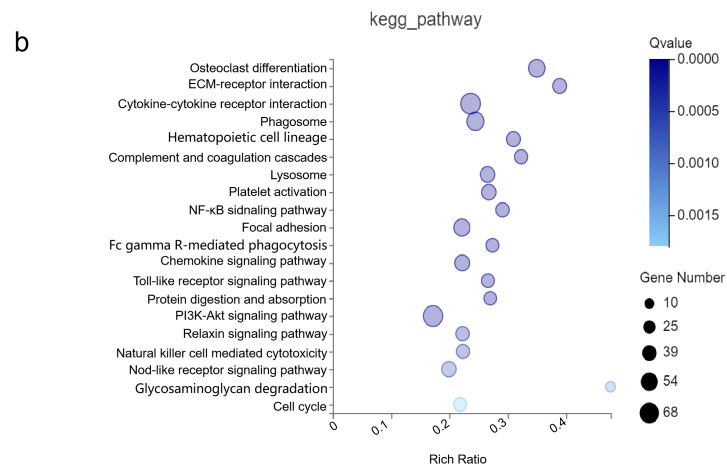

Supplement: Supplementary Materials — Supplementary_Figure_1: (a) The classification of component, process, and function (MCAO and BYHW). The y-axis represents the Gene Ontology (GO) annotation categories and the x-axis represents the number of total matched genes from a specific category. (b) KEGG pathway (MCAO and BYHW). X axis is enrichment score, and the y-axis indicates the KEGG pathway. The bubble size indicates the number of genes matched in the KEGG pathway. Supplementary_Figure_2: (a) The classification of component, process, and function (MCAO and NXT). (b) KEGG pathway (MCAO and NXT). Supplementary_Figure_3: (a) The classification of component, process, and function (MCAO and YYTN). (b) KEGG pathway (MCAO and YYTN). [file 4016935.f1.zip › Supplementary_material_1.pdf]

Supplementary material Fig.2

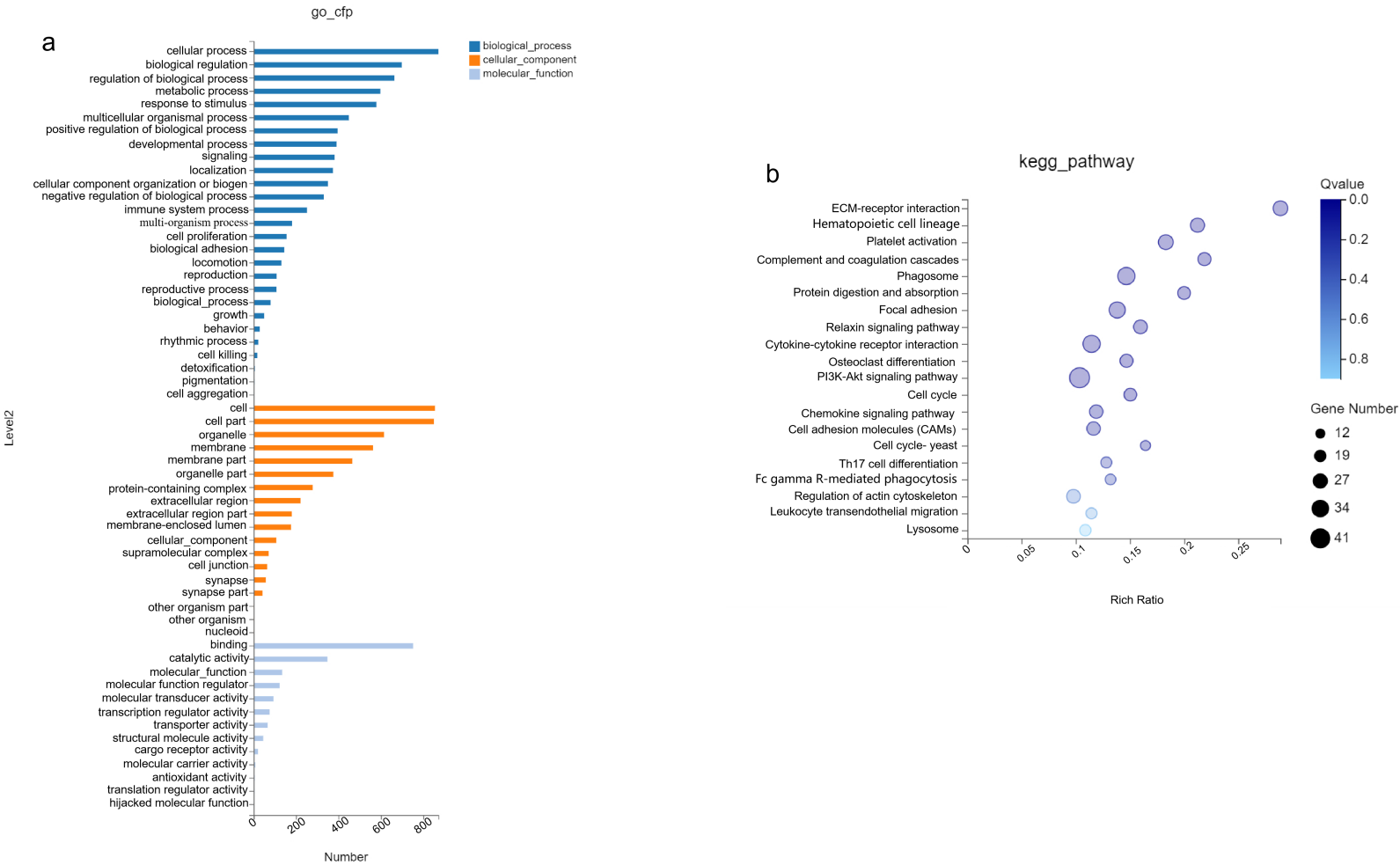

Supplement: Supplementary Materials — Supplementary_Figure_1: (a) The classification of component, process, and function (MCAO and BYHW). The y-axis represents the Gene Ontology (GO) annotation categories and the x-axis represents the number of total matched genes from a specific category. (b) KEGG pathway (MCAO and BYHW). X axis is enrichment score, and the y-axis indicates the KEGG pathway. The bubble size indicates the number of genes matched in the KEGG pathway. Supplementary_Figure_2: (a) The classification of component, process, and function (MCAO and NXT). (b) KEGG pathway (MCAO and NXT). Supplementary_Figure_3: (a) The classification of component, process, and function (MCAO and YYTN). (b) KEGG pathway (MCAO and YYTN). [file 4016935.f1.zip › Supplementary_material_2.pdf]

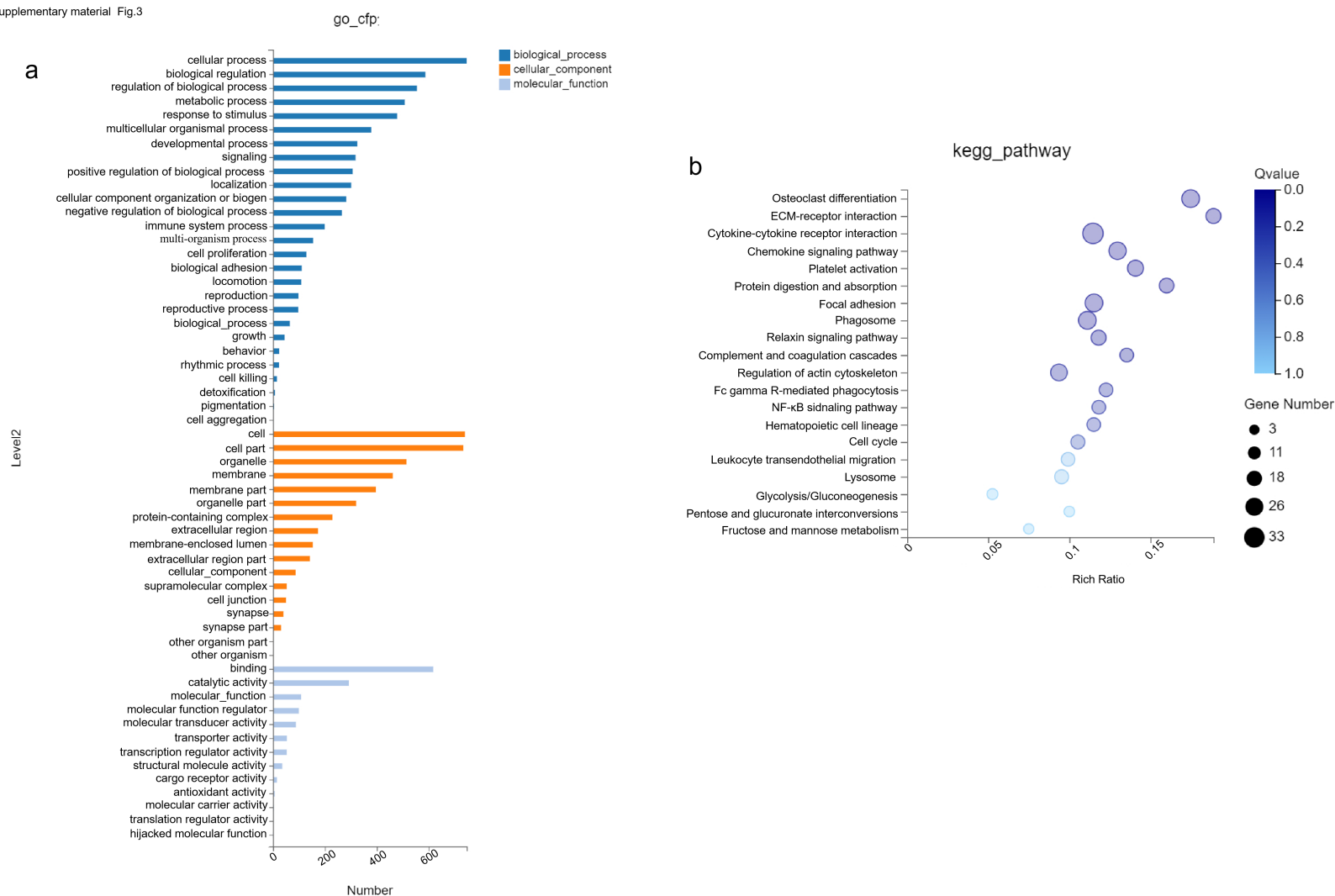

Supplement: Supplementary Materials — Supplementary_Figure_1: (a) The classification of component, process, and function (MCAO and BYHW). The y-axis represents the Gene Ontology (GO) annotation categories and the x-axis represents the number of total matched genes from a specific category. (b) KEGG pathway (MCAO and BYHW). X axis is enrichment score, and the y-axis indicates the KEGG pathway. The bubble size indicates the number of genes matched in the KEGG pathway. Supplementary_Figure_2: (a) The classification of component, process, and function (MCAO and NXT). (b) KEGG pathway (MCAO and NXT). Supplementary_Figure_3: (a) The classification of component, process, and function (MCAO and YYTN). (b) KEGG pathway (MCAO and YYTN). [file 4016935.f1.zip › Supplementary_material_3.pdf]
